# Something old, something new: Evolution of Colombian weedy rice (*Oryza* spp.) through de novo de‐domestication, exotic gene flow, and hybridization

**DOI:** 10.1111/eva.12955

**Published:** 2020-04-09

**Authors:** Verónica Hoyos, Guido Plaza, Xiang Li, Ana L. Caicedo

**Affiliations:** ^1^ Departamento de Agronomía Universidad Nacional de Colombia Bogotá Colombia; ^2^ Departamento de Agronomía Universidad Nacional de Colombia Bogotá Colombia; ^3^ Plant Biology Graduate Program University of Massachusetts Amherst MA USA; ^4^ Biology Department University of Massachusetts Amherst MA USA

**Keywords:** agricultural weed evolution, genotyping by sequencing, *Oryza sativa*, red rice

## Abstract

Weedy rice (*Oryza* spp.) is a worldwide weed of domesticated rice (*O. sativa*), considered particularly problematic due to its strong competition with the crop, which leads to reduction in yields and harvest quality. Several studies have established multiple independent origins for weedy rice populations in the United States and various parts of Asia; however, the origins of weedy rice in South America have not been examined in a global context. We evaluated the genetic variation of weedy rice populations in Colombia, as well as the contributions of local wild *Oryza* species, local cultivated varieties, and exotic *Oryza* groups to the weed, using polymorphism generated by genotyping by sequencing (GBS). We found no evidence for genomic contributions from local wild *Oryza* species (*O. glumaepatula*, *O. grandiglumis*, *O. latifolia,* and *O. alta*) to Colombian weedy rice. Instead, Colombian weedy rice has evolved from local *indica* cultivars and has also likely been inadvertently imported as an exotic pest from the United States. Additionally, weeds comprising de novo admixture between these distinct weedy populations now represent a large proportion of genomic backgrounds in Colombian weedy rice. Our results underscore the impressive ability of weedy rice to evolve through multiple evolutionary pathways, including in situ de‐domestication, range expansion, and hybridization.

## 1 INTRODUCTION

The creation of the agricultural environment through crop domestication provided a new environment for opportunistic plants. Thus in the last 10,000 years, humans have had to contend with the continued evolution of agricultural weeds, which are currently a leading cause of crop losses worldwide (Oerke, 2006). Understanding how agricultural weeds arise and evolve is necessary to devise effective weed management strategies (Vigueira, Olsen, & Caicedo, 2013). Particularly intriguing from an evolutionary point of view are agricultural weeds that are related to crop species. How plant groups amenable to domestication through artificial selection can also give rise to plants that compete with crops and evade human efforts of removal has long been a question of interest, and may provide insight into mechanisms of plant adaptation (Ellstrand et al., 2010; Vigueira et al., 2013).

Among weeds related to crops, weedy rice (*Oryza* spp.) stands out in its worldwide occurrence and its impact on crop rice (*Oryza sativa* L.), which is the staple food for about half of the world's population (Prasad, Shivay, & Kumar, 2017). Weedy rice is a type of weedy *Oryza* that only infests cultivated rice fields, and is particularly difficult to control in systems employing direct seeding and continuous sowing. Around the world, weedy rice is known to be one among the most problematic and predominant weeds of cultivated rice (Singh et al., 2017; Watson, 2002). The negative effects of weedy rice include yield reduction, harvest quality deterioration, rice milling quality decrease, crop seed contamination, production cost increase, and land devaluation (Clavijo & Montealegre, 2010; Delouche et al., 2007). The importance and negative impact of weedy rice has been reported in many regions of the world, including Europe (Fogliatto, Vidotto, & Ferrero, 2012; Grimm, Fogliatto, Nick, Ferrero, & Vidotto, 2013; Messeguer, Marfà, Català, Guiderdoni, & Melé, 2004), Latin America (Arrieta‐Espinoza et al., 2005; Avila, Marchezan, & Menezes, 2000; Canal, Arnaude, Ortiz‐Domínguez, Valverde, & Fuentes, 2009; Clavijo & Montealegre, 2010; Federici et al., 2001), the United States (Burgos, Norman, Gealy, & Black, 2006; Delouche et al., 2007), and Asia (Cao, Li, Song, Cai, & Lu, 2007; Cao et al., 2006; Zhiwen et al., 2016). Weedy rice infestations have been found to have the capacity to reduce cultivated rice yields by up to 80% (Olsen, Caicedo, & Jia, 2007).

While it has often not been well‐defined taxonomically (Kraehmer, Jabran, Mennan, & Chauhan, 2016), in many countries weedy rice is classified in the same genus and species as cultivated rice. In fact, a distinguishing feature of weedy rice is that it presents similar morphological and physiological characteristics to those of the crop, as well as similar nutritional and agro‐climatic requirements (Ellstrand et al., 2010; Valverde, 2005). However, unlike the crop, it has negative characteristics such as seed shattering (dispersal), red pericarp and associated seed dormancy, rapid growth, and efficient resource use, which make it a very competitive and successful plant (Delouche et al., 2007).

The occurrence of weedy rice in multiple and different regions of the world suggests parallel evolutionary events with independent origins. In fact, in recent years, genomic studies have been clarifying the extent to which weedy rice is an example of convergent evolution. Thus, for example, weedy rice from Northeast Asia has been found to have evolved from *japonica* rice varieties, which are the typical cultivars grown in colder regions in subtropical and temperate zones (Vigueira et al., 2019). Some weedy rice populations in South Asia and in South‐East Asia have evolved from *indica* cultivars, which are typically cultivated in lowland tropical and subtropical regions (Huang et al., 2017; Song, Chuah, Tam, & Olsen, 2014). Other weedy populations from South Asia have been found to have origins from *aus* cultivated varieties, which are typically grown in Bangladesh, or from local wild rice (*O. rufipogon* Griff., the ancestor of cultivated rice) (Huang et al., 2017, 2018). The origin of weedy rice in different regions of the world is thus often influenced by the prevailing local variety and/or the availability of reproductively compatible wild *Oryza* (Cao et al., 2006; Ishikawa et al., 2005; Olsen et al., 2007).

In the world, Colombia ranks 26th among rice‐producing countries and is third in Latin America after Brazil and Peru. Rice is the fourth most important crop in Colombia, after sugarcane, oil palm, and banana. However, rice cultivation in Colombia has reported losses from 30% to 73% due to weeds (Cobb & Reade, 2010). A major weed of cultivated rice in Colombia is weedy rice, for which the presence of 24 plants per m^2^ in early‐growth stages (40 days after emergence) can reduce crop yields by 50% (Fischer & Ramírez, 1993). Despite its economic importance, no systematic survey of the evolutionary origins of weedy rice has yet been carried out in Colombia.

Several *Oryza* types could be possible sources of weedy rice in Colombia. The main varieties of cultivated rice grown are routinely released by Fedearroz (National Federation of Rice Growers) and are classified morphologically as *indica.* A few areas in the western part of the country, collectively known as “Bajo Cauca,” are also cultivated with traditional landraces (i.e., “criollos”), whose classification is unknown. Additionally, Colombia and other countries of Central and South America are unique in the occurrence of several native species of wild *Oryza*, different from those found in Asia, and these could play a role in weedy rice evolution. These wild species are as follows: *O. glumaepatula* Steud. (also known as *O. glumipatula* Steud., diploid, AA genome, 2n = 24, and included in the *O. sativa* complex), and *O. latifolia* Desv., *O. grandiglumis* (Döll) Prod., and *O. alta* Swallen (all tetraploid, with CCDD genomes, 2n = 48, and included in the *O. officinalis* Wall. complex.) (Veasey et al., 2011). In Colombia, native wild *Oryza* species grow mainly in patches of untreated vegetation bordering commercial rice crops; they are found in humid soils, wetlands, and streams, soils with high organic matter content, and can also appear in zones distant from agricultural areas (Villafañe, Estrada, Lentini, Fory, & Palacio, 2007). *Oryza latifolia* is the species with the greatest distribution, whereas *O. grandiglumis* has the most restricted range (Estrada, Villafañe, Palacio, Fory, & Lentini, 2010). As with most wild *Oryza* species, this South American *Oryza* has traits that are often found in weedy rice, such as red pericarp color, shattering, and seed dormancy.

The plethora of possible contributors to weedy rice in Colombia make the country an interesting case study to contrast with weedy rice evolution in other world regions. Understanding how weedy rice emerges in and adapts to agricultural environments is a necessary first step for designing control strategies for this pest (Vigueira et al., 2013). To determine the origins of weedy rice in Colombia, we have carried out a thorough sampling of weedy rice diversity in the country, as well as sampling of local cultivars and wild species. Here, we use genome‐level information to understand the relationships between Colombian weedy rice and local *Oryza.* We further examine the origins of Colombian weedy rice within an international context, to attempt to understand how the weed evolves and diversifies at a global scale.

## 2 MATERIALS AND METHODS

### 2.1 Plant material and DNA extraction

**FIGURE 1:**
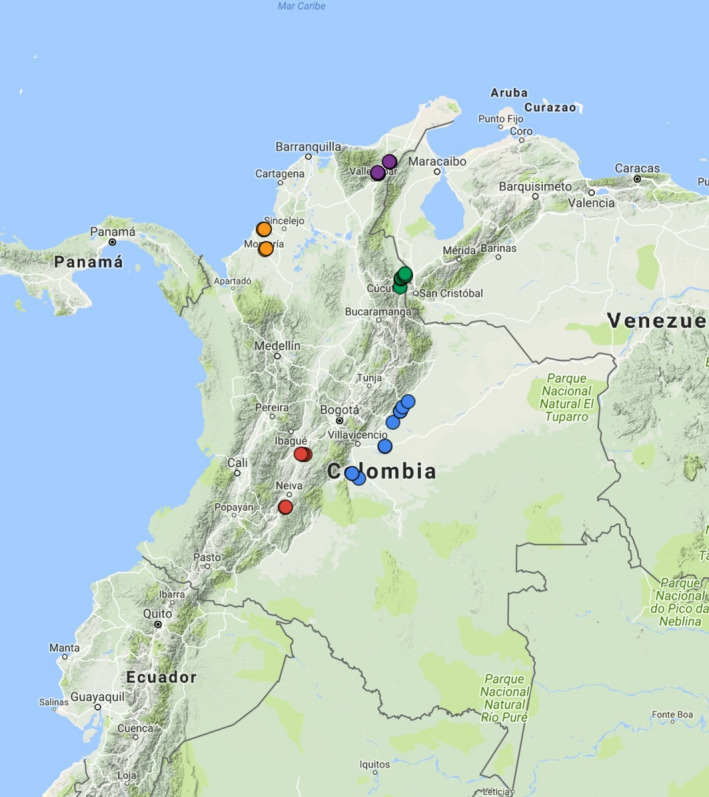
Map of Colombia showing weedy rice collection areas used in this study. Central zone (red dots), Llanos plains (blue dots), Bajo Cauca river valley (orange dots), North coast (purple dots), and Santanderes area (green dots)

Weedy rice seeds were collected by sampling in the five principal rice production areas of the country in 2014–2015 (Figure 1). Twenty‐six accessions were collected in the Central zone, 34 in the Llanos plains, 28 in the Bajo Cauca river valley, 26 in the North coast, and 26 in the Santanderes area, for a total of 140 Colombian weedy rice (CWR) accessions. These were additionally classified according to hull color (lemma and palea), pericarp color, grain size, and awn presence, abundance, and length. Nine varieties of commercial rice were supplied by the National Federation of Rice Growers (Fedearroz), which included the following: four varieties that are currently cultivated and have been in the market for 10–17 years (F473 cultivated in the low Cauca valley area, F2000 in Santanderes and North coast zones, F174 in the Eastern plains, and F60 in the Central zone), one variety with national relevance and cultivated for approximately 17 years (F50), and four varieties that have left the market but were important over a 25‐year period (Cica 6, Cica 8 and Cica 9 and Orizica1). Additionally, 19 landraces (traditional crop with manual dry seeding system, present in the low Cauca valley area) that belong to the germplasm bank of the Fedearroz Monteria section were sampled. We also obtained seeds of 22 wild South America *Oryza* from the International Rice Research Institute (IRRI); these included seven *O. glumaepatula*, six *O. grandiglumis*, five *O. alta*, and four *O. latifolia* (Table S1).

Staggered sowing of all selected materials (CWR, commercial rice and landraces) was carried out in the Weed Science greenhouse at Universidad Nacional de Colombia. In order to break dormancy, weeds were exposed to 50°C for 12 hr (materials that did not germinate under this treatment were kept at 50°C for 72 hr). South American wild rice materials were planted in the Biology greenhouse at the University of Massachusetts, following a dormancy breaking treatment at 50°C for 5 days. 200 mg of young leaf tissue was used for DNA extraction with the Qiagen DNeasy^®^ Plant Mini Kit. The concentration and purity of the DNA in the samples was quantified with a Qubit 2.0 Fluorometer; 30 μl of DNA with a concentration of 30–100 ng/μl for each sample was sent to the Cornell University Biotechnology Resource Center (BRC) for genotyping by sequencing (GBS).

### 2.2 GBS library preparation and sequence analysis

Genotyping by sequencing was used to detect polymorphisms distributed throughout the genome among our samples (Elshire et al., 2011). Restriction digestions were carried out with the enzyme *Ape*KI, and the fragments were ligated with individual barcoded and common adapters. DNA fragments were pooled for further PCR amplification to enrich the libraries. Single‐end fragments of 100 base pairs (bp) were sequenced on an Illumina HiSeq 2500 platform. Raw GBS data have been deposited in the NCBI SRA (experiment SUB6244405). Initial data processing was performed at the Biotechnology Institute of Cornell University with the TASSEL‐GBS v.3.0 pipeline (Glaubitz et al., 2014) and the MSU7 rice reference genome. The first filters removed SNPs with minimum minor allele frequency <0.01 or missing data per site >90%. Additional filters were applied to the initial VCF file with the NGSEP pipeline (Duitama et al., 2014) at the University of Massachusetts. Final SNPs were supported by at least five reads (for the analysis of Colombian material) or three reads (for the global analysis), and displayed heterozygosity levels of <50%. SNPs with more than 15% missing data and individuals with more than 70% missing data were removed. Only biallelic SNPs were retained, filtering any other type of variants. The final VCF has been deposited in DRYAD.

Data generated for Colombian and South American samples were integrated with other published GBS databases that included 128 cultivars, 173 weedy rice samples, and 53 samples of the wild ancestor of cultivated rice (*O. rufipogon*/*O. nivara*) from Asia (Huang et al., 2017; Vigueira et al., 2019); nine cultivars and 17 weedy rice samples from the United States (Burgos et al., 2014); and four out‐group samples (*O. meridionalis* and *O. barthii*) from these same datasets (NCBI Short Read Archive experiment SRX576894), for a total of 574 accessions (Table S1).

### 2.3 Population analyses

Population structure was analyzed using STRUCTURE (version 2.3.3, Hubisz, Falush, Stephens, & Pritchard, 2009), on the Massachusetts Green High Performance Computing Cluster (http://www.mghpcc.org/). Due to the limitation in the amount of input data that can be handled by the program (Falush, Stephens, & Pritchard, 2003; Pritchard, Stephens, & Donnelly, 2000), approximately 10,000 SNPs were randomly selected for each analysis with a roughly 15,000 base pairs (bp) spacing. Heterozygotes were recorded as “N,” and all the simulations were run with data coded as haploid, because weedy and cultivated rice are highly self‐pollinated. The program was run for an ancestral “*admixture*” model, with no correlated allele frequencies. Runs were carried out using K values between 1 and 15, with three replicates per K, a burn‐in period of 100,000 and 500,000 subsequent iterations. The optimal number of genetic groupings was determined using ΔK (Evanno, Regnaut, & Goudet, 2005) according to the program Structure Harvester (Earl & vonHoldt, 2012). The program CLUMPP (Jakobsson & Rosenberg, 2007) was used to obtain a single Q matrix for each K. The final matrix for each K value was visualized with Distruct (Rosenberg, 2004). For comparison, we also analyzed our complete SNP dataset for worldwide samples with the Bayesian clustering analysis fastStructure (version 1.0, Raj, Stephens, & Pritchard, 2014), without recoding heterozygotes, with no prior grouping. FastStructure runs were conducted for K from 2 to 8. The best K was determined through chooseK.py, and the POPHELPER R package was used to generate an image (Francis, 2017).

To investigate the genetic divergence among individuals for all SNPs, the program SmartPCA from the EIGENSOFT package (Patterson, Price, & Reich, 2006; Price et al., 2006) was used. Figures with eigenvalues as coordinates were generated by RStudio 1.0.143.

To infer the phylogenetic relationships among samples, RAxML (Randomized Axelerated Maximum Likelihood) version 8 (Stamatakis, 2014) was used. The RAxML HPC2 on XSEDE tool was selected in the CIPRES portal (http://www.phylo.org/), with GTRGAMMA model and 1,000 bootstraps. Because SNP data only present variable sites, ascertainment bias correction (ASC) was performed. The best phylogenetic tree result was plotted using iTol v4 (Letunic & Bork, 2016).

Genetic diversity for each population was measured by evaluating the expected heterozygosity calculated for all loci, and paired F_ST_ was used to estimate the genetic differences among populations with the software ARLEQUIN (ver 3.5.2.2., Excoffier & Lischer, 2010). Additionally, an AMOVA was performed to analyze variation among and within populations.

### 2.4 Shattering (*sh4*) and pericarp color (*Rc*) gene sequencing

Seventy‐one weedy rice accessions from the five rice production areas in Colombia, five commercial rice, and ten landraces, for a total of 96 accessions, were selected for candidate gene analysis. Additionally, for the *Rc* gene, four accessions of wild diploid materials (*O. glumaepatula*) were sequenced, and for *sh4,* publicly available sequences of *O. glumaepatula* (GU221016, GU221017, and DQ421813) were included for comparison.

A portion of the second exon of *sh4* gene was amplified using primers designed with Primer3 (Untergasser et al., 2012), based on the PopSet by Thurber et al. (2010) (sh4_01_for: 5′‐ACGGGCACCTGACTGCTACG‐3′; sh4_01_rev: 5′‐GAGGTGGGTGGTGGTGATGG‐3′), yielding a band size of ~678 bp. PCR was performed in a 25 μl volume containing 2 μl of genomic DNA template (5 ng/μl), 4 µl of dNTPs (2.5 mM), 0.50 µl of each primer (10 µM), 12.5 µl of 2X GC Buffer I, 0.25 µl of TaKaRa LA Taq DNA polymerase (5 U/µl; Takara, Shiga, Japan), and 0.50 µl of DMSO. PCR amplification was carried out by initial denaturation at 94°C for 3 min, followed by 30 cycles of denaturation at 94°C for 30 s, 61°C annealing for 30 s, and 72°C elongation for 2 min, and a final extension of 72°C for 5 min.

Exon 7 of *Rc* was amplified with primers designed off the PopSet by Gross et al. (2010), Rc_02_for (5′‐AGTGGCATCACCTGAAAATACC‐3′) y Rc_02_rev (5′‐CGGCTTTATAGAAATAGAGGGAGT‐3′), yielding a band size of ~509 bp. PCR amplifications were carried out in a 20 μl reaction mixture containing 2 μl of template DNA (5 ng/µl), 1.6 μl of dNTPs (2.5 mM), 1 μl of each primer (10 µM), 2 μl of 10X *Ex Taq* Buffer, and 0.1 μl of TaKaRa Ex Taq (5 U/µl). The PCR program consisted of an initial denaturing step at 95°C for 3 min, followed by 30 cycles of denaturation 95°C for 1 min, annealing at 54°C for 30 s, and extension at 68°C for 30 s, with a final extension at 72°C for 10 min.

Sanger sequencing was carried out by either GENEWIZ or Beckman. DNA sequences were aligned and edited with the software Geneious (ver 9.1.3., http://www.geneious.com, Kearse et al., 2012). Sequence alignments have been deposited as PopSets in NCBI GenBank. *Rc* sequences were examined for the presence/absence of the 14‐bp deletion present in exon 7 associated with loss of pericarp color; *sh4* sequences were examined for the occurrence of the G/T single nucleotide substitution in exon 2 associated with loss of shattering during domestication. Gene trees were constructed under the Tamura‐Nei genetic distance model and the neighbor‐joining (NJ) method in the program Geneious. For the *sh4* gene, three *O. glumaepatula* DNA sequences deposited in the GenBank, accessions GU221016 and GU221017 reported by Thurber et al. (2010) and DQ421813 reported by Li, Zhou, and Sang (2006), were included. Trees were plotted in iTol v4 (Letunic & Bork, 2016).

Seed shattering and pericarp color were assessed for sequenced accessions. The determination of pericarp color was visually performed at the mature stage of the grain taking into account the intensity of the color. When there were doubts about the color due to the differences in weedy rice maturity in the field, the 2% potassium hydroxide (KOH) test was performed (Louisiana State Seed Testing Laboratory, 1980; Rosta, 1975), immersing dehulled seeds in this solution for 10–20 min. After this time, the KOH solution turns red or dark orange if the seeds are red, while the white seed shows a light golden yellow color or remains colorless. Shattering measures were taken 30 days after flowering using a digital force gauge to measure breaking tensile strength (BTS), and averaging values for 10 seeds in the same panicle (Thurber et al., 2010). Statistical analysis was performed by analysis of variance (ANOVA) of one factor, using the statistical package SPSS version 23. The comparison of means was performed by the Tukey test at *p* < .05.

## 3 RESULTS

### 3.1 Colombian weedy rice origins

**FIGURE 2:**
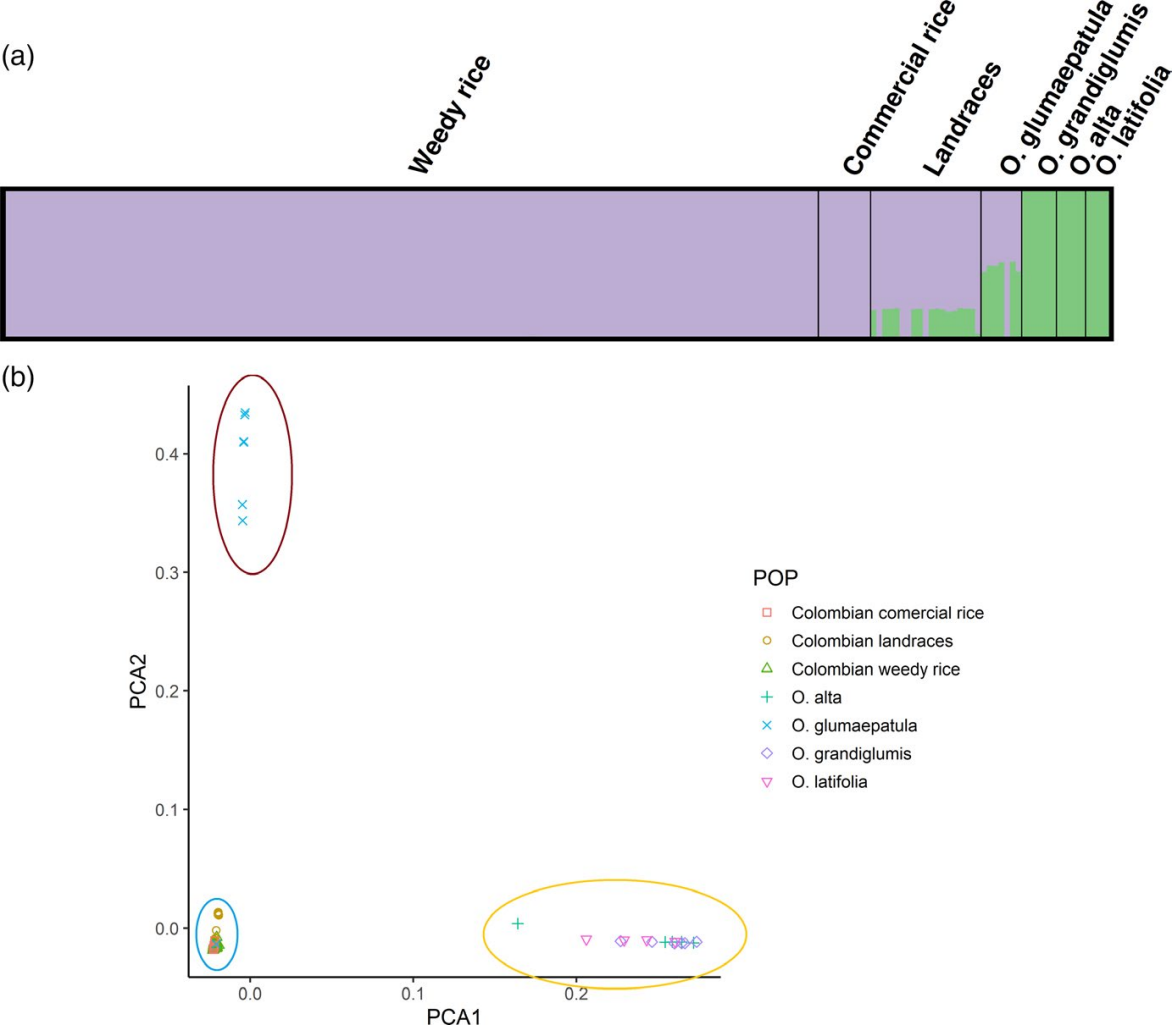
Population analysis of 190 *Oryza* accessions (140 Colombian weedy rice, 9 commercial rice lines from Colombia, 19 landraces from Colombia, 7 *O. glumaepatula*, 6 *O. grandiglumis*, 5 *O. alta,* and 4 *O. latifolia*). (a) Population structure, each individual is represented by a color bar, with color partitions that reflect the relative proportion of genetic membership in a given group (*K* = 2). (b) Principal component analysis

A total of 47,200 high‐quality SNPs were obtained from GBS for the 190 Colombian and wild South American *Oryza* samples. When all materials are included, a population structure analysis based on 11,808 SNPs suggests the presence of two differentiated populations (highest ΔK at *K* = 2; Evanno et al., 2005; Figure 2a; Tables S2 and S3). One population includes all Colombian weedy rice (CWR) and commercial rice varieties; the second corresponds to native tetraploid wild *Oryza* species (*O. grandiglumis*, *O. alta,* and *O. latifolia*). Landraces and *O. glumaepatula* appear as a mixture of these populations. These results are congruent with a principal component analysis (PCA) on all SNPs, which shows separate clusters for tetraploid wild *Oryza* species with CCDD genome (*O. grandiglumis*, *O. alta,* and *O. latifolia*), the diploid wild species (*O. glumaepatula*), and CWR with commercial rice and landraces (Figure 2b, Table S4).

**FIGURE 3:**
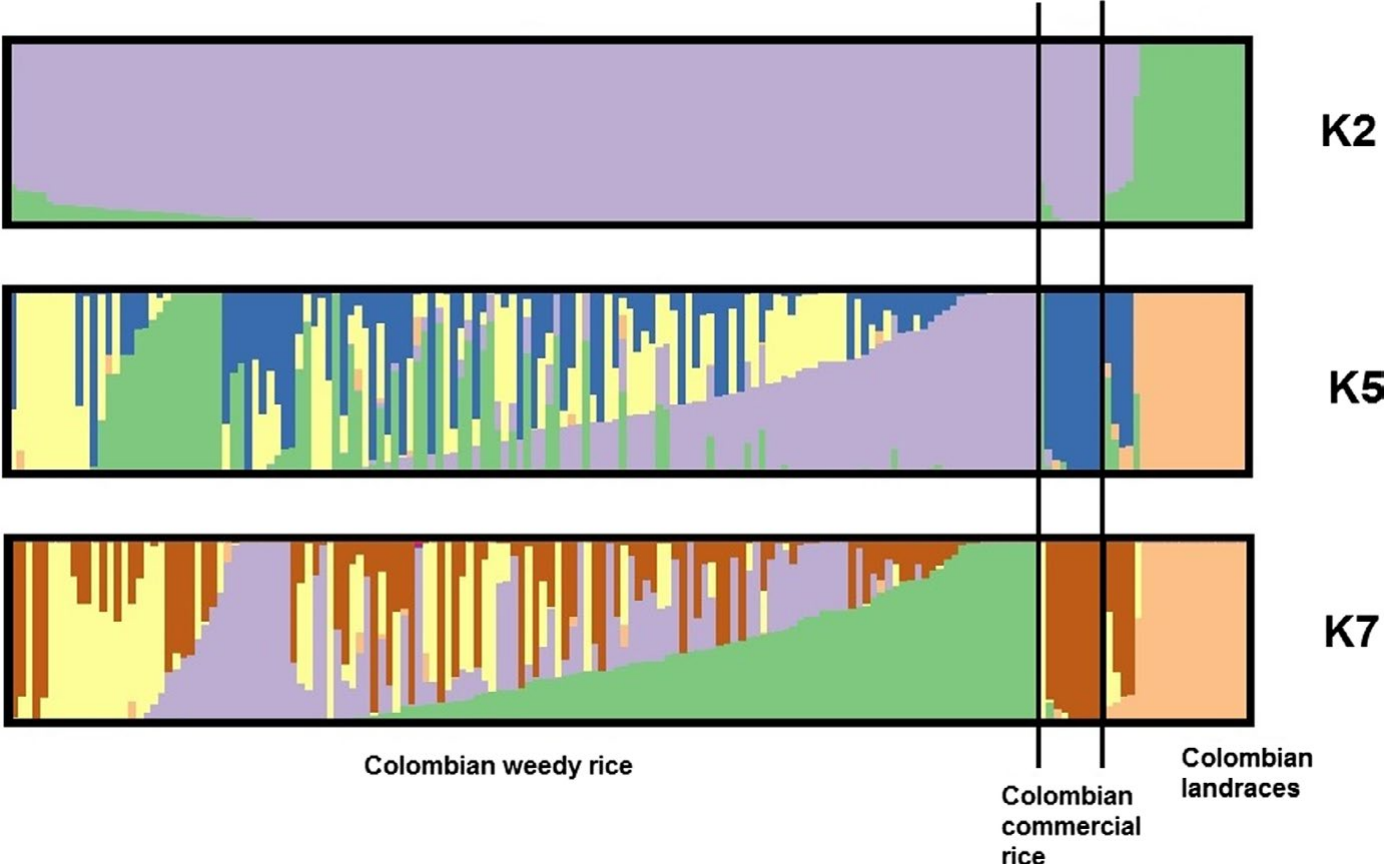
Population structure of 168 Colombian *Oryza* accessions (140 weedy rice, 9 commercial rice, and 19 landraces). Each individual is represented by a color bar, with color partitions that reflect the relative proportion of genetic membership in a given group (*K* = 2, *K* = 5, and *K* = 7)

Both population structure and PCA suggest no contribution of native wild *Oryza* to CWR; thus, we excluded these groups and re‐analyzed population structure with the available 10,923 SNPs. Three ΔK peaks were observed, at *K* = 2, *K* = 5, and *K* = 7, with the highest occurring at *K* = 7 (Figure 3, Tables S5 and S6). Under the progressive models, Colombian landraces are highly differentiated from CWR and commercial rice, and the commercial varieties make contributions to a heterogeneous collection of CWR samples. These analyses suggest that the sources of remaining CWR genomic heterogeneity are from unsampled material, perhaps from sources outside of Colombia.

**FIGURE 4:**
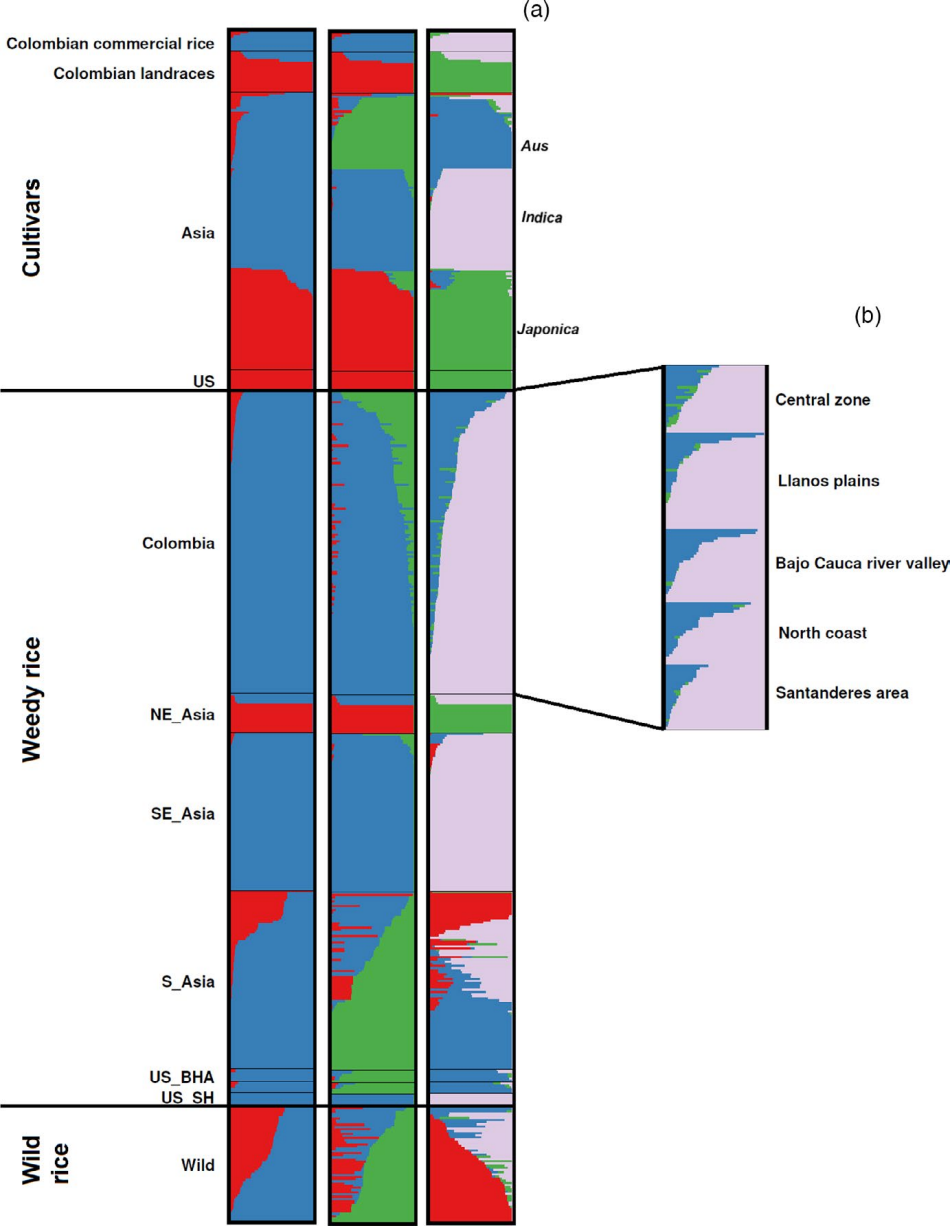
Population structure of 548 Colombian and global *Oryza* accessions. Each individual is represented by a colored bar, with color partitions that reflect the relative proportion of genetic membership in a given group. (a) Results of the analysis of Colombian accessions (140 weedy rice, 9 commercial rice, and 19 landraces), Asian (128 cultivars, 173 weedy rice, and 53 wild rice), and United States (9 cultivars and 17 weedy rice) at *K* = 2, *K* = 3, and *K* = 4 populations. (b) Population structure of Colombian weedy rice according to the rice geographic areas at K = 4

To further investigate the origin of CWR, we obtained high‐quality SNPs from merged data from this study and from published GBS data of global samples of weedy rice, cultivated rice, and the wild ancestor of cultivated rice, a species complex known as *O. rufipogon/nivara*, and carried out a population structure analysis on 12,284 SNPs. We excluded wild rice from South America and out‐groups. The highest Δ*K* value was obtained for *K* = 2, with also substantially high values for *K* = 3–4 (Figure 4a, Tables S7 and S8). At *K* = 2, *indica* + *aus* and *japonica* cultivated lineages are clearly differentiated, showing that CWR and Colombian commercial varieties most resemble *indica* cultivars, a common cultivar group in the tropical lowlands of Asia, while most Colombian landraces used in the last 400 years resemble *japonica*. Because the Evanno method can underestimate *K* when there is a hierarchical structure of the population (Waples & Gaggiotti, 2006), we examined models with *K* = 3 and *K* = 4. These models are consistent population structure detected for US and Asian weedy rice (Huang et al., 2017; Reagon et al., 2010; Vigueira et al., 2019) and readily distinguish the three main groups of cultivated groups of rice, *indica*, *aus,* and *japonica*, with *K* = 4 also distinguishing the wild ancestor of cultivated rice (Figure 4a). With this higher resolution, we see that CWR has admixture, with contributions primarily from *indica* and *aus* cultivated varieties (and very occasionally from *japonica*; Figure 4), and shares resemblance with other weed groups around the world. A fastStructure run based on 21,664 SNPs, for which a *K* value of 5 maximized the marginal likelihood, gave congruent results (Fig. S1, Table S9). Based on these results, we classified all CWR samples according to predominant ancestry (>80% contribution from a single group), resulting in 5 *aus*‐like individuals, 85 *indica*‐like individuals, and 50 admixed *indica‐aus* individuals. Interestingly, these CWR types seem distributed in all the rice‐growing regions of Colombia (Figure 4b).

**FIGURE 5:**
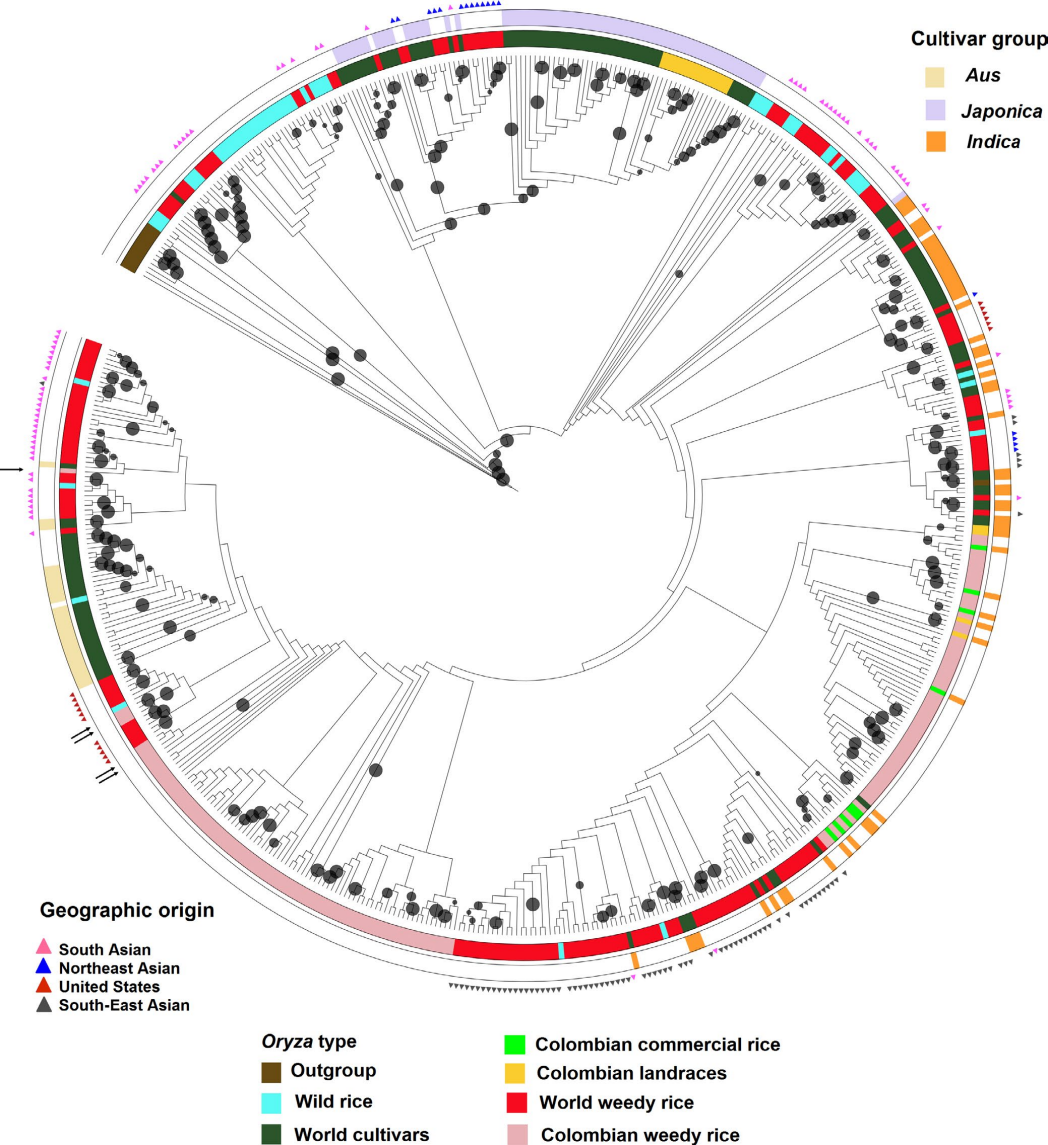
Maximum‐likelihood tree of 559 Colombian and global *Oryza* accessions and out‐groups. Circles represent the level of bootstrap support of each branch (only bootstrap values >70% are shown). Inner ring colors indicate *Oryza* type groups, as indicated in the figure key. Outer ring colors represent cultivar groups of *O. sativa*. Colored triangles at the tip of each branch represent the geographic origin for world weedy rice accession. Arrows show accessions of CWR identified as >80% *aus*‐like. Individual accession IDs can be found in Figure S1

The ancestral contributions in CWR could stem directly from local (Colombian) cultivars that have undergone de‐domestication, or from dispersal of weedy rice from other world regions into Colombia. We constructed a phylogenetic tree with our global set of samples, including out‐groups, and note CWR falling in different portions of the tree (Figure 5 and Figure S2). In particular, CWR classified as *indica*‐like based on our structure analysis is related to world weedy rice with known *indica* origins (e.g., US SH weeds, South Asian weeds) and to known *indica* cultivars. However, the closest relationship is with the Colombian commercial rice varieties, suggesting that local de‐domestication events gave rise to *indica*‐like weedy rice in Colombia. Of the five accessions of CWR identified as >80% *aus*‐like based on our population structure analysis, two are nested within the BHA clade, which is a monophyletic group of US weeds with known *aus* origin (Figure 5). Two others cluster close by, but with low bootstrap support, and one clusters with *indica*‐like weeds from South Asia, also with low bootstrap support. This suggests that *aus*‐like weeds in Colombia are more likely to have originated directly from US weeds and perhaps were inadvertently imported, although an importation event from Asia cannot be completely discounted for some accessions. The low incidence of non‐admixed *aus*‐like weeds in Colombia compared to the United States makes an origin in Colombia with export to the United States unlikely. CWR samples identified as admixed in our population structure analyses fall between known *indica* and *aus* cultivars and do not group with *aus*‐like weeds from South Asia, consistent with their likely origins though hybridization events between *aus*‐like and *indica*‐like weeds in Colombia (Figure 5).

### 3.2 Genetic diversity in Colombian weedy rice

We examined levels of genetic diversity in CWR compared to other *Oryza* groups. Colombian weedy rice as a whole has lower levels of expected heterozygosity than any cultivar group examined (Table 1). Among CWR groups, *aus*‐like weeds present a greater genetic diversity, despite their low numbers, than *indica*‐like or weeds of admixed origins (Table 1). When genetic diversity was analyzed for the different rice‐growing regions in Colombia, we did not see a substantial difference among regions (Table 1).

**TABLE 1:** Genetic diversity according to *Oryza* groups, Colombian weedy rice ancestry, and geographic area

*Oryza* groups	*H*_e_	Ancestry	*H*_e_	Geographic area	*H*_e_
Colombian weedy rice (CWR)	0.146	*indica*	0.170	Central	0.188
		*aus*	0.367	Llanos plains	0.168
		Admixed *indica*‐*aus*	0.209	Bajo Cauca river valley	0.198
				North coast	0.199
				Santanderes	0.182
Landraces	0.197	–	–	–	–
Commercial rice	0.263	–	–	–	–
*japonica*	0.161	–	–	–	–
*indica*	0.188	–	–	–	–
*aus*	0.212	–	–	–	–

Note

*H*_e_: expected heterozygosis calculated for all loci.

We assessed levels of genetic differentiation between CWR groups and major cultivated rice groups, which confirmed the very low *F*_ST_ values for *indica‐*like weeds and commercial rice from Colombia (*F*_ST_ = 0.093), which is of *indica* type, followed by an also modest *F*_ST_ for *indica*‐like CWR and *indica* (*F*_ST_ = 0.162) (Table S10). Additionally, the lowest *F*_ST_ values for Colombian *aus*‐like weeds and crop groups were with *aus* cultivars (*F*_ST_ = 0.232). Comparisons of CWR with weedy rice groups distributed worldwide revealed that the lowest levels of differentiation for CWR *aus*‐like weeds were with US weeds of *aus* origin (BHA; *F*_ST_ = 0.14; Table S11). This F_ST_ value was lower than that between *aus*‐like weeds and *aus*, supporting a direct origin from US weeds. Interestingly, the lowest F_ST_ values for admixed CWR were in comparison with *indica*‐like CWR, supporting the greater genetic contribution of this group to these admixed weeds.

### 3.3 Shattering and the *sh4* gene

**FIGURE 6:**
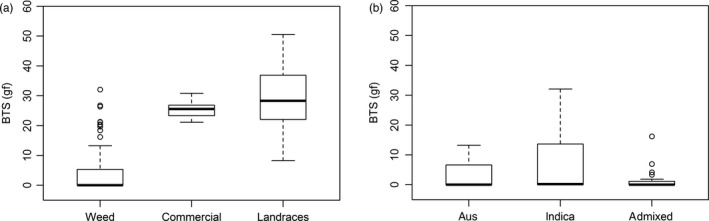
Seed shattering phenotype. Distributions are the mean values of breaking tensile strength (BTS) expressed in grams‐force (gf). The black line represents the median of each distribution. (a) Shattering accessions of Colombian weedy rice, commercial varieties, and landraces. (b) Shattering accessions of weedy rice according to their ancestry

Seed shattering was recorded for 86 accessions of CWR and cultivated varieties (commercial and landraces) as reported in Hoyos, Plaza, and Caicedo (2019). In general terms, weeds have lower BTS (equivalent to greater shattering) necessary to separate grains from the panicle with a general average of 4.6 gf (±8.3), followed by Colombian commercial varieties with an average of 25.5 gf (±3.7), and finally landraces with 30.8 gf (±13.1) (Figure 6a). Shattering averages were significantly different (Tukey's test [HSD] at the 0.05 probability level) across CWR ancestral groups (Table S12), with *indica*‐like weeds displaying less seed shattering (BTS 6.9 gf ± 10.0), and admixed *indica‐aus* weeds showing the greatest shattering (BTS 1.4 gf ± 3.5) (Figure 6b).

Sequencing of 628 bp within the second exon *of sh4* for 87 accessions (69 weedy rice, 5 commercial varieties, 10 landraces, and 3 *O. glumaepatula*) revealed that all CWR samples, regardless of origin, showed the presence of the T allele associated with the weakening of the gene function and loss of shattering during domestication (Figure S3). The fixation of the derived T allele occurs despite the primarily shattering phenotype displayed by CWR (Figure S3, Table S13).

### 3.4 Pericarp color and the *Rc* gene

**FIGURE 7:**
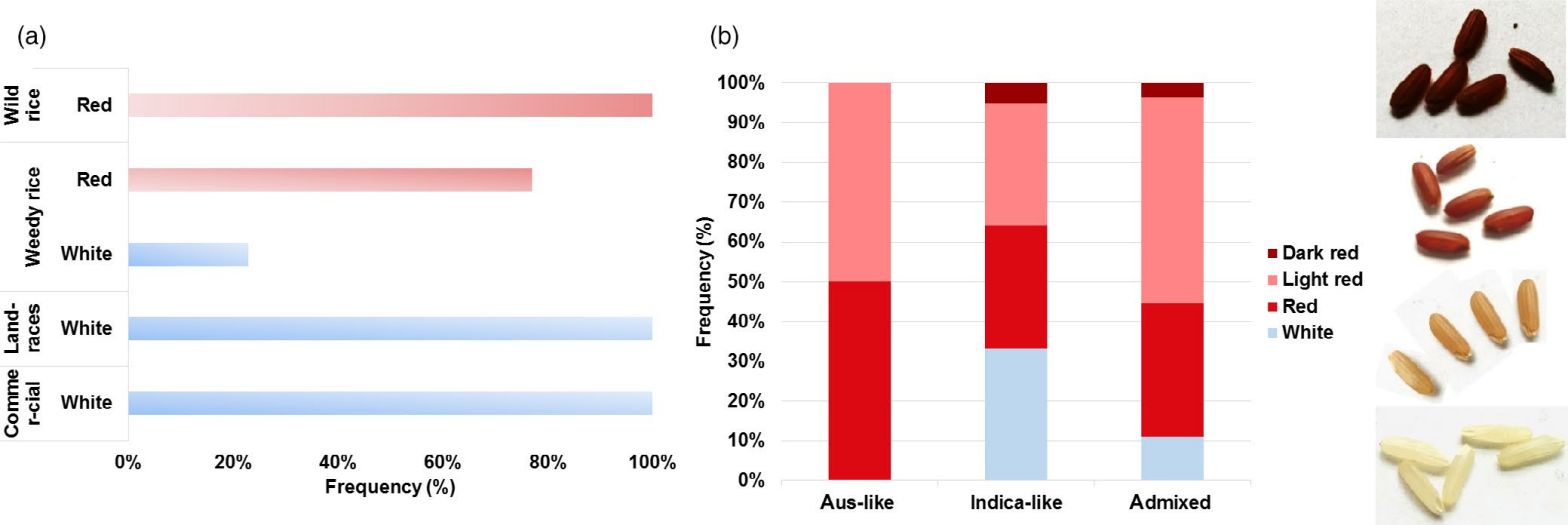
Pericarp color phenotype. (a) Percentage of individuals of Colombian wild rice, weedy rice, commercial varieties, and landraces with white and red colors. (b) Percentage of weedy rice accessions according to their ancestry with white and red colors

We recorded pericarp color for 95 accessions, 70 Colombian weedy rice, 5 commercial varieties, 10 landraces, and 4 *O. glumaepatula* (Figure 7a, Table S14). All domesticated rice was found to have white pericarps, whereas all wild *O. glumaepatula* have red pericarps. In contrast, 77% of CWR samples had red pericarps, with the remainder being white. Additionally, we observed variation in the extent of pericarp pigmentation among weeds, with light red being present in 52% of these samples. The extent of pericarp coloration in CWR was correlated with ancestry, with 100% of *aus*‐like weeds possessing red pericarps, whereas *indica* and admixed weeds have some samples with white pericarps (33% and 11%, respectively) (Figure 7b).

We sequenced 421 bp spanning the seventh exon of the *Rc* locus for phenotyped accessions. As expected, all accessions with white pericarp (both weeds and cultivated rice) possessed the 14‐bp deletion, which has been shown to be responsible for the loss of color in most cultivated rice (Furukawa et al., 2007; Sweeney, Thomson, Pfeil, & McCouch, 2006; Figure S4). For accessions with red pericarps, wild diploid samples of *O. glumaepatula* lacked the deletion, as expected, as did most of the red pericarp CWR, independent of their ancestry. However, seven weedy rice accession (mainly of *indica* origin) also with red pericarps carried the 14 bp deletion as well. We examined for the presence of the two additional mutations which have been reported to reverse the effect of the 14 bp deletion on *Rc* function, re‐establishing the red colored pericarp (Brooks, Yan, Jackson, & Deren, 2008; Lee, Lupotto, & Powell, 2009), but neither were found in the accessions evaluated. Our results suggest that in a subset of weeds, other loci are having an effect on pericarp color.

## 4 DISCUSSION

### 4.1 Colombian weedy rice has multiple origins

Understanding the origin and evolution, genetic diversity, and structure of weedy rice populations is basic knowledge that is necessary for the design of effective methods of weed control (He, Kim, & Park, 2016; He et al., 2014; Vigueira et al., 2013), as well as preventing weedy rice adaptation to crop fields (Huang et al., 2017). Three main hypotheses have at various times been put forward for the evolution and origins of weedy rice: (a) direct colonization and adaptation of wild rice groups to the cultivated environment; (b) "de‐domestication" of cultivated rice varieties that become weedy; and (3) gene flow between cultivated rice and reproductively compatible wild populations, leading to weedy rice of hybrid origins (Vigueira et al., 2013). In studies of weedy rice around the world, examples of all of these origins have been found (e.g., Huang et al., 2017; Li, Li, Jia, Caicedo, & Olsen, 2017; Reagon et al., 2010; Song et al., 2014; Vigueira et al., 2019), underscoring the ease with which weedy rice can repeatedly arise in diverse cultivated environments.

As a continent with a unique set of wild *Oryza* species, some of which are reproductively compatible with *O. sativa*, South America presents an interesting case study for the origins of local weedy rice. In Colombia, many of these wild *Oryza* species can be found growing at the borders of rice fields. Our population structure and PCA results, however, show no contribution of wild South American species nor of wild Asian species (*O. rufipogon* or *O. nivara*) to Colombian weedy rice. These results are consistent with microsatellite‐based reports that weedy rice from southern Brazil groups separately from *O. glumaepatula*, which is native to South America, and also from *O. rufipogon*, *O. longistaminata*, and *O. glaberrima*, which are wild AA species from Asia (Goulart, Borba, Menezes, & Merotto, 2014). The difference in ploidy among wild *Oryza* of the CCDD type and cultivated rice with an AA genome likely creates a genetic barrier that reduces the possibility of gene flow between most South American wild species and cultivated rice (Oka & Chang, 1961). The remaining wild species, *O. glumaepatula*, has the same AA genome as cultivated rice, and there are reports of it hybridizing with *O. sativa* (Espinoza & Lentini, 2005). However, there is no evidence for hybridization in our study, and we suggest that in several rice‐growing regions of Colombia, *O. glumaepatula* is too geographically limited to have an effect. Although this species grows in Colombia in non‐intervention areas bordering commercial rice crops (Estrada et al., 2010), it has a low potential distribution in rice‐growing regions of the Caribbean and inter‐Andean valleys (Villafañe et al., 2007). Early studies with microsatellites in Colombia reported that some CWR accessions from Tolima (the Central zone) with black hulls and awns showed high genetic similarity with *O. rufipogon* (González et al., 2003, 2007); given the lack of evidence for contribution of this Asian species to CWR, we suggest that this was an erroneous conclusion likely due to the lack of inclusion of the diversity present in rice cultivars, in particular exclusion of *aus* varieties.

Multiple studies have supported de‐domestication as the principal route for weedy rice origins around the world, for example, in Bhutan (Ishikawa et al., 2005), Brazil (Goulart et al., 2014), United States (Li et al., 2017; Reagon et al., 2010), China (Cao et al., 2006; Li et al., 2017; Qiu et al., 2014; Zhang et al., 2008; Zhang, Dai, Wu, Song, & Qiang, 2012), Malaysia (Song et al., 2014), Korea (He et al., 2016), and South‐East Asia (Vigueira et al., 2019). De‐domestication is an evolutionary process that involves a loss of some traits added under domestication, and likely begins with the formation of a "volunteer weed," a crop that acquires a trait by mutation or hybridization, which provides an advantage in the environment (Ellstrand et al., 2010; Gressel, 2005).

In Colombia, two types of cultivated rice are planted, either of which could potentially give rise to weedy rice through de‐domestication. Landraces, commonly known as “criollos,” are unimproved varieties cultivated in the department of Chocó and the Low Cauca area that are planted manually in small plots mainly for self‐consumption (Espinal, Martínez, & Acevedo, 2005; Pérez, Saavedra, Chimá, & Toro, 2005). Our results are consistent with prior results from chloroplast DNA polymorphisms (Villafañe, Bocanegra, & Moreno, 2010) in finding that most Colombian landraces are *japonica* cultivars. The influence of *japonica* in the country has a possible origin with the European colonization of the Americas and the introduction of rice; some historians believe that Christopher Columbus brought this crop on his second trip in 1,493 to Central and South America (GRiSP, 2013). However, these *japonica* landraces are not the source of weedy rice in Colombia.

Commercial cultivars in Colombia have long been presumed to be *indica*, which is confirmed by our results. These cultivars also seem to be the largest source of weedy rice in Colombia, as most of our sampled CWR was found to be *indica*‐like, clustered close to Colombian cultivars in our tree, and harbored very little genetic differentiation with Colombian commercial cultivars based on *F*_ST_. Our results support growing evidence that in many parts of the world, the origins of this weed are often related to local crop varieties. However, research on weedy rice in the United States has also shown that de‐domesticated weeds do not necessarily always evolve from local cultivars, as SH and BHA weedy rice descend from *indica* and *aus* cultivars, respectively, neither of which are cultivated commercially in the United States (Gealy, Agrama, & Eizenga, 2009; Londo & Schaal, 2007; Reagon et al., 2010). Such exotic origins of de‐domesticated weeds are also evident in Colombia, which harbors a small proportion of weedy rice that is *aus*‐like in genomic background. Yet, there are no records of *aus* cultivars ever having been grown in Colombia, and its cultivation is restricted to the center and Northeast of India and Bangladesh (Garris, Tai, Coburn, Kresovich, & Mccouch, 2005; Londo, Chiang, Hung, Chiang, & Schaal, 2006), which are the centers of origin of this cultivar (Civáň, Craig, Cox, & Brown, 2015).

The most likely explanation for the contribution of *aus* cultivars to CWR is through an involuntary introduction of BHA weedy rice from the United States. This is supported by the phylogenetic clustering of most *aus*‐like CWR and US BHA weeds, and the low genetic differentiation (as measured by *F*_ST_) between CWR and US weeds with the same ancestry. To our knowledge, rice seeds in Colombia have not been sourced from Asia historically, and another possibility that BHA weeds arose from Colombian *aus*‐like weedy rice is unlikely given that Colombia has not exported rice to the United States or contributed to its breeding program. BHA weedy rice was possibly introduced to the United States as a stock seed contaminant or as escaped breeding material (Delouche et al., 2007; Reagon et al., 2010). Similarly, it has been previously suggested that weedy rice in Latin America and the Caribbean was introduced as a contaminant in rice seeds imported from the United States (Hernandez, 1971; Ortiz‐Dominguez, 1999). While this is an unlikely explanation for *indica*‐like CWR, it is the most likely explanation for the occurrence of *aus*‐like CWR.

The last, and second most common, group of CWR seems to be an admixture of *indica* and *aus* backgrounds. Given the occurrence of both *indica*‐like weeds and *aus*‐like weeds in the country, the most likely explanation for the origin of these admixed weeds is hybridization between weedy groups in Colombian fields. Admixed CWR is thus the product of a complex evolutionary scenario consisting of three parts: (a) a de novo de‐domestication event (local *indica* cultivars giving rise to *indica*‐like CWR), (b) exotic gene flow (exotic de‐domesticated *aus*‐like weed arrives from elsewhere), and (c) a hybridization event (*indica*‐*aus* weeds arise through hybridization between the two weedy rice groups). In hybridizing, a widely successful new type of weed, admixed *indica‐aus* CWR, has emerged, which is now found in all rice‐growing regions of Colombia, and which rivals its progenitor weeds in abundance.

That all three groups of Colombian weedy rice have managed to adapt to the principal rice‐growing regions in Colombia is shown by the lack of geographic structure in the weeds. This adaptation has occurred, in spite of what are relatively lower levels of expected heterozygosity than observed in the commercial and landrace varieties used in the country. The rice‐growing regions in Colombia vary in environmental conditions such as temperature, altitude, and rainfall, and rice is planted at different times throughout the year. However, there is great homogeneity in the provenance of the seeds used, because approximately 80% of the commercial varieties sown in the country are supplied by a single company, FEDEARROZ, and in some areas, this percentage exceeds 90% (personal communication FEDEARROZ 2017). Additionally, the exchange of contaminated seeds among farmers from different regions could be another factor that affects homogeneity in the regions. For the year 2016, only 58.8% of the planted area in Colombia was sown with weed‐free‐certified seed (DANE & FEDEARROZ, 2017). This implies that the remaining areas are being planted with seed that may not be weed free and that often stems from farmers exchanging seed saved from their fields, providing a route for weedy rice to expand its range.

### 4.2 The source of weedy traits in Colombian weedy rice

As for all de‐domesticated weeds, a major question about weedy rice is how it has acquired the traits that enable weediness, which are not typical of their crop ancestors. Two particularly characteristic traits of weedy rice are the dispersal of seeds through shattering and the red pericarp color, which is associated with seed dormancy. As expected, Colombian weeds showed high levels of seed shattering in comparison with cultivated varieties, but also high phenotypic variability in this trait, similar to other weed rice studies (Markus, Merotto, Barcelos, & Dalazen, 2013; Nunes, Delatorre, & Merotto, 2014; Nunes, Markus, Delatorre, & Merotto, 2014; Thurber et al., 2010). The greatest shattering levels were observed in admixed *indica‐aus*, while the lowest values were found in *indica*‐like weeds. Similar results were found in South Asian weeds, where *indica*‐like weeds showed lower shattering (higher BTS) compared to *aus*‐like or wild type (Huang et al., 2017).

All CWR groups carry the domesticated allele of the gene *sh4,* which contains the non‐shattering T substitution that was fixed during rice domestication. *Sh4* is involved in the formation and function of the abscission zone from which a mature grain separates from the pedicel (Li et al., 2006). While there is evidence that cultivated alleles of *sh4* are not always sufficient for loss of shattering and are likely dependent on genetic background (Zhu, Ellstrand, & Lu, 2012), *sh4* remains among the most significant genes to have experienced selection by humans during domestication (Li et al., 2006; Wang et al., 2018; Zhang et al., 2009). Similar *sh4* allele frequencies have been observed for other de‐domesticated weedy rice in the United States (Thurber et al., 2010), Japan (Akasaka, Konishi, Izawa, & Ushiki, 2011), other parts of Asia (Huang et al., 2018; Zhu et al., 2012), and Italy (Grimm, 2014). Thus, the results for CWR add to mounting evidence that de‐domesticated weeds evolve the shattering trait through novel genetic mechanisms rather than reversals to ancestral alleles. Preliminary mapping studies in SH and BHA weedy rice groups suggest that these novel loci may not necessarily be shared among independently arisen weedy rice groups (Qi et al., 2015; Thurber, Jia, Jia, & Caicedo, 2013). Given that a subset of CWR shares ancestry with BHA weeds from the United States, however, it is possible that the same loci are involved in reacquisition of shattering in BHA as in Colombian *aus*‐like and *indica‐aus* admixed weeds groups.

The proanthocyanidins responsible for pericarp are believed to play an important role in seed dormancy and in the protection against bacteria and fungi in the soil (Sweeney et al., 2007). In weedy rice, seed dormancy allows the seeds to remain in the soil for long periods of time, guaranteeing the permanence of the group (Grimm, 2014). As is common in weedy rice groups (Ziska et al., 2015), most of Colombian weedy rice accessions (77%) have reddish pericarp coloration. These reddish colorations in the pericarp are present in all types of Colombian weedy rice (*aus*, *indica* and *admixed*). In contrast, weeds with white pericarps, which are a minority, only occur in *indica*‐like or admixed weeds, supporting the perceived advantage of red pericarps in weedy rice.

As expected based on prior studies of *Rc* (Furukawa et al., 2007; Sweeney et al., 2007), the red pericarp in most CWR can be explained by the presence of the functional ancestral *Rc* alleles that lack the 14‐bp deletion that knocks out the gene and leads to white pericarps. *Rc* is a pleiotropic gene that affects both pericarp coloration and dormancy through promotion of expression of genes involved in the biosynthesis of abscisic acid, which is a dormancy‐inducing hormone, and through activation of a conserved network of eight genes involved in synthesis of flavonoids, which are pigments (Gu et al., 2011). The occurrence of wild functional *Rc* alleles in what is a de‐domesticated weed is most likely explained through direct inheritance of this low‐frequency allele from domesticated ancestors. Although most cultivated rice has a white pericarp and the 14‐bp deletion in *Rc*, cultivars with red pericarps and ancestral alleles also exist, and this standing variation in crop ancestors could have been selectively favored in weedy rice. Although no red pericarp cultivars occur in Colombian commercial rice, we cannot be absolutely sure these have not been used for breeding purpose in the past.

A startling find is that a small subset of weedy rice display red pericarps despite having the 14‐bp *Rc* deletion. Restorer mutations in this gene have previously been reported in some cultivars, such as *Rc‐r* (Lee et al., 2009) and *Rc‐g* (Brooks et al., 2008), but we did not find these. Our data are insufficient to explain whether undiscovered restorer mutations have occurred or if other loci are involved in this red coloration, but this finding again underscores the selective advantage that red pericarps must confer on weedy rice.

## 5 CONCLUSIONS

Since the beginning of agriculture, weedy plants have evolved to flourish in crop fields with detrimental effects on agricultural production. Understanding the source of such plants and how they adapt to agricultural conditions are important pre‐requisites for ameliorating their impact crop yields. As studies of weedy rice, a major weed of cultivated rice, accumulate, it has become evident that this weed has had multiple independent origins, most often from domesticated ancestors. Our results from Colombian weedy rice support this growing evidence that the origins of weedy rice are influenced more by domesticated groups than by wild rice, even when local wild *Oryza* species exist, as several do in South America. De‐domestication has additionally been increasingly recognized as a significant mechanism for agricultural weed origins beyond the weedy rice system (Ellstrand et al., 2010), as evidenced by recent studies showing crop origins for Tibetan semi‐wild wheat and weedy rye (Burger & Ellstrand, 2014; Burger, Holt, & Ellstrand, 2007; Jiang et al., 2019).

An important consequence of this study is the highlighting of the need to consider weedy rice evolution in any locality within a global context. Although *indica*‐like weedy rice in Colombia most likely has origins in de‐domestication from the local *indica* crop, our data suggest that *aus*‐like weeds are likely exotic introductions, a finding that would have been impossible to discern if we had not compared our Colombian weeds with the range of worldwide diversity in weedy and cultivated *Oryza*. Our study also underscores how dynamic the evolution of weedy rice can be: Given a local weedy rice origin and an exotic introduction, a new category of admixed weedy rice has successfully emerged in the country. We propose that continued use of a global perspective in weedy rice evolution studies, as well as in other weed evolution studies, will enhance our understanding of the facility with which agricultural weeds can evolve and the mechanisms they use to adapt to the agricultural environment.

## CONFLICT OF INTEREST

No conflicts of interest have been declared.

## Supporting information

Fig S1

Fig S2

Fig S3

Fig S4

Table S1‐S14

## Data Availability

Raw genotyping‐by‐sequencing data have been deposited at the NCBI Short Read Archive (experiment BioProjectIDPRJNA616397). DNA sequences obtained for this study have been deposited in GenBank under accessions MT089735—MT089829 for *Rc* gene and MT089830—MT089913 for *sh4* gene. VCF files have been submitted to DRYAD (10.5061/dryad.sf7m0cg2q).
