# Supplementary figures and images for: Something old, something new: Evolution of Colombian weedy rice (Oryza spp.) through de novo de‐domestication, exotic gene flow, and hybridization
Source: Evol Appl. 2020 Apr 9;13(8):1968–83. doi: 10.1111/eva.12955 (PMC7463356; doi:10.1111/eva.12955)

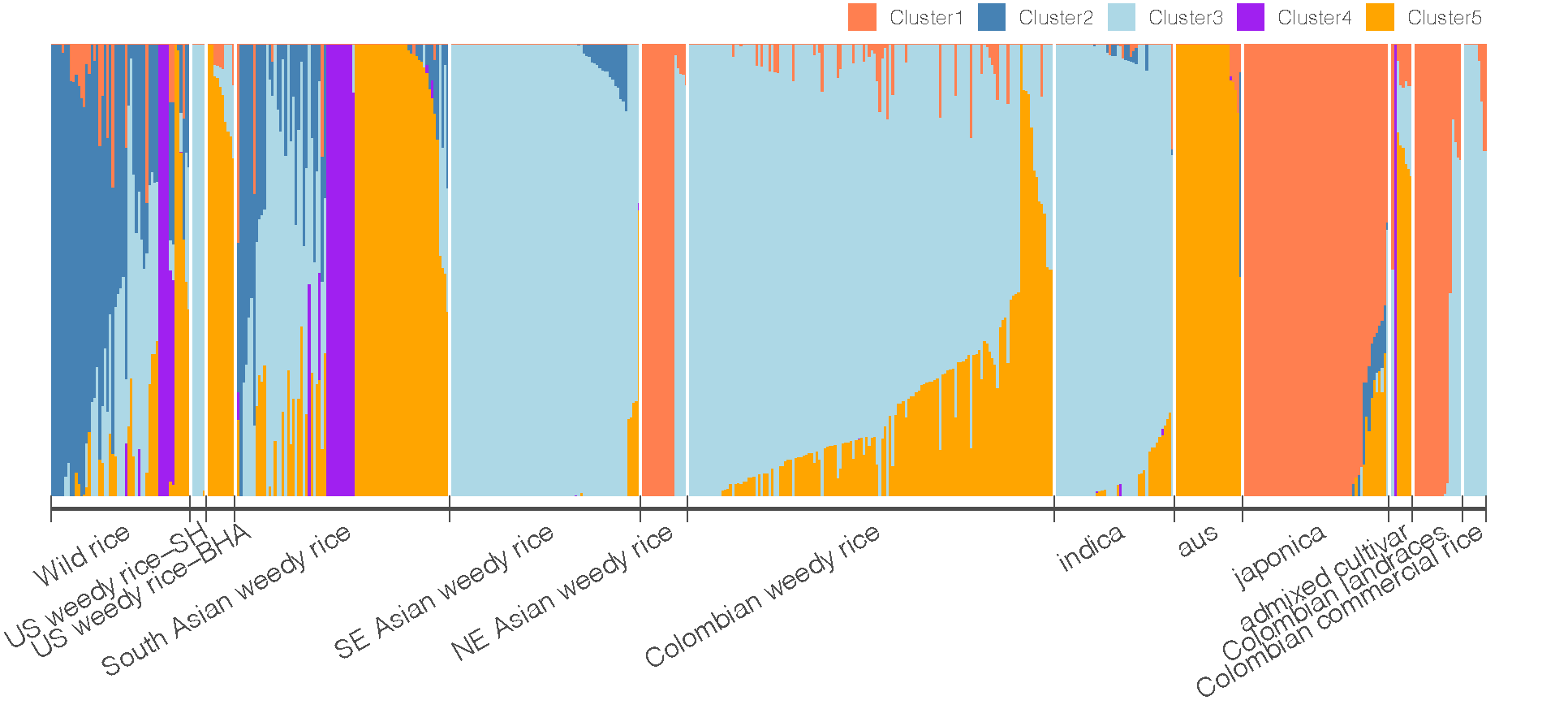

Supplement: Supplementary file 1 — Fig S1 [file EVA-13-1968-s001.tif]

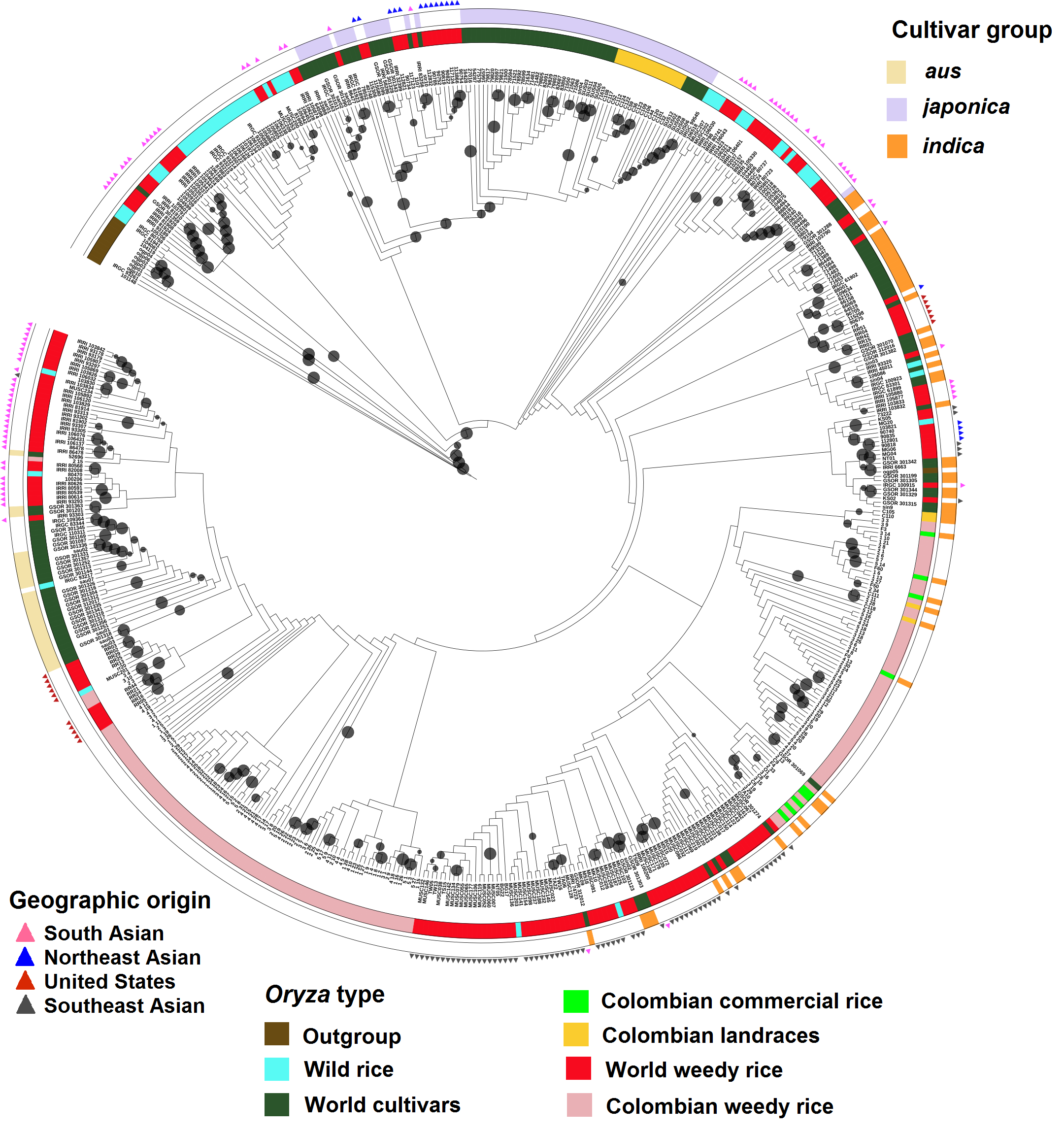

Supplement: Supplementary file 2 — Fig S2 [file EVA-13-1968-s002.tif]

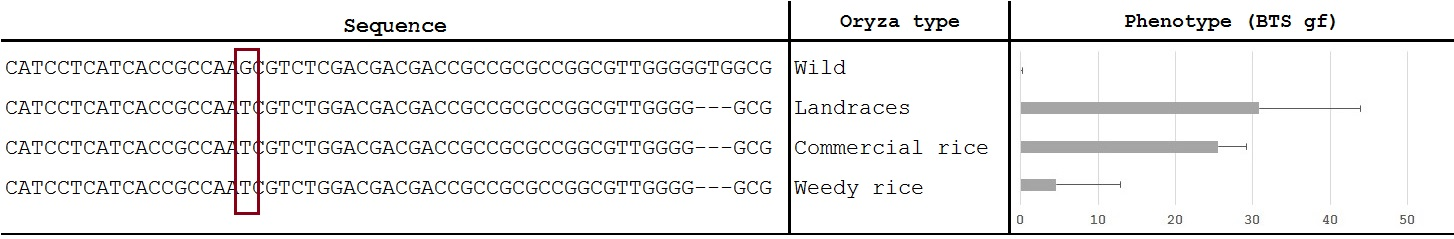

Supplement: Supplementary file 3 — Fig S3 [file EVA-13-1968-s003.tif]

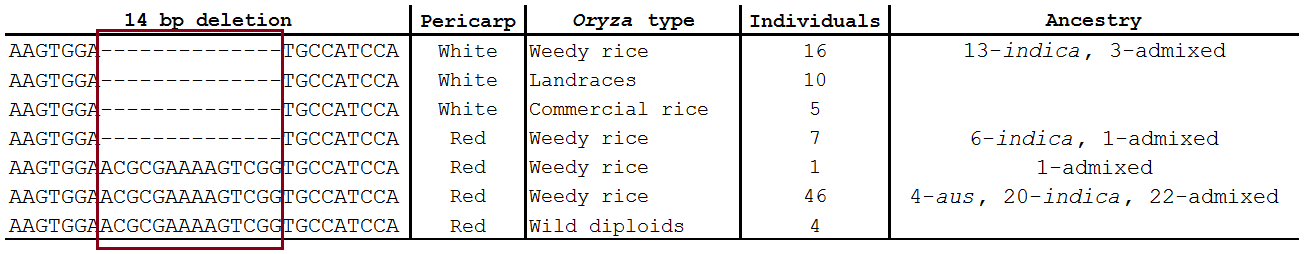

Supplement: Supplementary file 4 — Fig S4 [file EVA-13-1968-s004.tif]
